# Modulation of Calcitonin, Parathyroid Hormone, and Thyroid Hormone Secretion by Electrical Stimulation of Sympathetic and Parasympathetic Nerves in Anesthetized Rats

**DOI:** 10.3389/fnins.2017.00375

**Published:** 2017-06-30

**Authors:** Harumi Hotta, Akiko Onda, Harue Suzuki, Philip Milliken, Arun Sridhar

**Affiliations:** ^1^Department of Autonomic Neuroscience, Tokyo Metropolitan Institute of GerontologyTokyo, Japan; ^2^Galvani BioelectronicsStevenage, United Kingdom

**Keywords:** superior laryngeal nerve, cervical sympathetic trunk, thyroid, parathyroid, calcitonin, parathyroid hormone, electrical stimulation

## Abstract

The thyroid and parathyroid glands are dually innervated by sympathetic (cervical sympathetic trunk [CST]) and parasympathetic (superior laryngeal nerve [SLN]) nerve fibers. We examined the effects of electrical stimulation of efferent or afferent nerve fibers innervating the thyroid and parathyroid glands on the secretion of immunoreactive calcitonin (iCT), parathyroid hormone (iPTH), 3,3′,5-triiodothyronine (iT3), and thyroxine (iT4) from the thyroid and parathyroid glands. In anesthetized and artificially ventilated rats, thyroid venous blood was collected. The rate of hormone secretion from the glands was calculated from plasma hormone levels, measured by ELISA, and the flow rate of thyroid venous plasma. SLNs or CSTs were stimulated bilaterally with rectangular pulses with a 0.5-ms width. To define the role of unmyelinated nerve fibers (typically efferent), the cut peripheral segments were stimulated at various frequencies (up to 40 Hz) with a supramaximal intensity to excite all nerve fibers. The secretion of iCT, iT3, and iT4 increased during SLN stimulation and decreased during CST stimulation. iPTH secretion increased during CST stimulation, but was not affected by SLN stimulation. To examine the effects of selective stimulation of myelinated nerve fibers (typically afferent) in the SLN, intact SLNs were stimulated with a subthreshold intensity for unmyelinated nerve fibers. iCT, iT3, and iT4 secretion increased during stimulation of intact SLNs at 40 Hz. These results suggest that excitation of myelinated afferents induced by low intensity and high frequency stimulation of intact SLNs promotes secretion of CT and thyroid hormones from the thyroid gland, potentially via reflex activation of parasympathetic efferent nerve fibers in the SLN.

## Introduction

The secretory functions of various endocrine glands, including those regulated by humoral factors, may be controlled by autonomic nerves (Kagitani et al., 2008; Uchida, 2015; Uchida and Kagitani, 2015). It is well known that circulating thyroid stimulating hormone (TSH) controls the secretion of 3,3′,5-triiodothyronine (T3) and thyroxine (T4), and calcium ions control the secretion of calcitonin (CT) and parathyroid hormone (PTH). In addition, the thyroid and parathyroid glands are innervated by parasympathetic and sympathetic nerves. The thyroid gland receives vagal innervation mainly via the superior laryngeal nerve (SLN; Nonidez, 1931), which contains both afferent and efferent fibers. An earlier study in our laboratory revealed a role of SLN efferents in thyroid blood flow, where electrical stimulation of cut peripheral SLN segments (with supramaximal strength for a period of 2 min) increased thyroid venous blood flow (Ito et al., 1987). This response increased in a frequency-dependent manner from 2 to 40 Hz via cholinergic and non-cholinergic mechanisms, predominantly at low and high frequency stimulation, respectively. Electrical stimulation of bilateral cervical sympathetic trunks (CSTs) at 10–80 Hz decreased thyroid blood flow (Ito et al., 1987).

The regulation of hormone secretion from the thyroid and parathyroid glands by autonomic nerves has been supported by previous histological findings (Grunditz et al., 1996), various *in vitro* and *in vivo* pharmacological studies (Cardinali and Stern, 1994), and *in vivo* denervation experiments (Cardinali and Stern, 1994; Stern et al., 1994). SLN activation by stimulation of a cut peripheral segment of the nodose ganglion (30 Hz, 20 s on/20 s off, for 15 min) increased the content of radiolabeled iodide in thyroid venous blood in dogs (Ishii et al., 1968). However, a systematic study of the effects of autonomic nerve stimulation on the secretion of CT and PTH from thyroid and parathyroid glands into the thyroid venous blood has yet to be completed.

The SLN is composed of myelinated and unmyelinated fibers (Hishida et al., 1997). In rats, the great majority of preganglionic autonomic fibers are unmyelinated, which is not the case in humans (Hedger and Webber, 1976; Domeij et al., 1989; Sato et al., 1996). Most myelinated fibers in the rat SLN (96–99%) are afferent fibers (Domeij et al., 1989). Activation of myelinated afferent Aδ fibers in the SLN (50 Hz, for 20 s) elicits tachycardia and an increase in blood pressure (Hanamori et al., 1996), while lower frequency electrical stimulation of the SLN (1–10 Hz, for 15 s) induced a reflex vasodilation of the rat hindlimb (Possas and Lewis, 1997). However, whether thyroid and parathyroid functions are influenced by stimulation of myelinated SLN fibers was unclear.

The first aim of the present study was to examine the effect of autonomic (both sympathetic and parasympathetic) efferent nerve stimulation at various frequencies on the secretion of CT and PTH, T3, and T4 from the thyroid and parathyroid glands in rats. For study aim 1, cut peripheral ends of CSTs and SLNs were electrically stimulated at the supramaximal strength of all nerve fibers. The second aim of our study was to examine whether the selective activation of myelinated SLN fibers influenced hormonal secretion. For study aim 2, intact SLNs were electrically stimulated at varying frequencies with an intensity lower than the threshold for unmyelinated fibers.

## Materials and methods

Nineteen male Sprague Dawley rats were used in this study (500–650 g, 4–7 months of age, purchased from Japan SLC, Inc.). Experiments were conducted in accordance with the Guidelines for Proper Conduct of Animal Experiments (established by the Science Council of Japan in 2006) and approved by the Animal Care and Use Committee of Tokyo Metropolitan Institute of Gerontology. The rats were used for three different experiments, as summarized in Table 1. To determine the effect of efferent nerve fiber activation, we stimulated the cut peripheral end of CSTs (*n* = 6) or SLNs (*n* = 6). We also stimulated intact SLNs (*n* = 7).

**Table 1:** Summary of the three experimental groups.

**Experiment group**	**Cut**	**Stimulation**	**Stimulus strength**	**Target fibers**	***n***
Group 1	CSTs	CSTs (cut peripheral ends)	0.5 ms, > 40T	Unmyelinated	6
Group 2	SLNs	SLNs (cut peripheral ends)	0.5 ms, > 40T	Unmyelinated	6
Group 3	Intact	SLNs (intact)	0.5 ms, 2T	Myelinated	7

*CST, cervical sympathetic trunk; SLN, superior laryngeal nerve*.

*T, The threshold intensity that evoked a twitch of the cricothyroid muscle*.

Experiments were performed under urethane anesthesia (initially 1.1 g/kg, intraperitoneally). Respiration was maintained by an artificial respirator (SN-480-7; Shinano Seisakusho, Tokyo, Japan) through a tracheal cannula. Artificial respiration was adjusted to sustain the end-tidal CO_2_, which was monitored by a gas monitor (Capnostream™20p; Oridion Medical, Jerusalem, Israel), at 3–4%. Systemic arterial blood pressure was monitored through a catheter in the femoral artery. Any necessary drugs and fluids were injected through a catheter in the femoral vein. The depth of anesthesia was routinely assessed by monitoring the animal's motion, respiration, blood pressure, and heart rate. If these conditions became unstable during the experiment, additional doses of urethane (0.11 g/kg, s.c. or i.v.) were administered. The body temperature was monitored by a rectal probe and maintained between 37.0 and 38.0°C by a direct current heating pad and infrared lamp (ATB-1100; Nihon Kohden, Tokyo, Japan).

### Stimulation of autonomic nerve fibers

**Figure 1:**
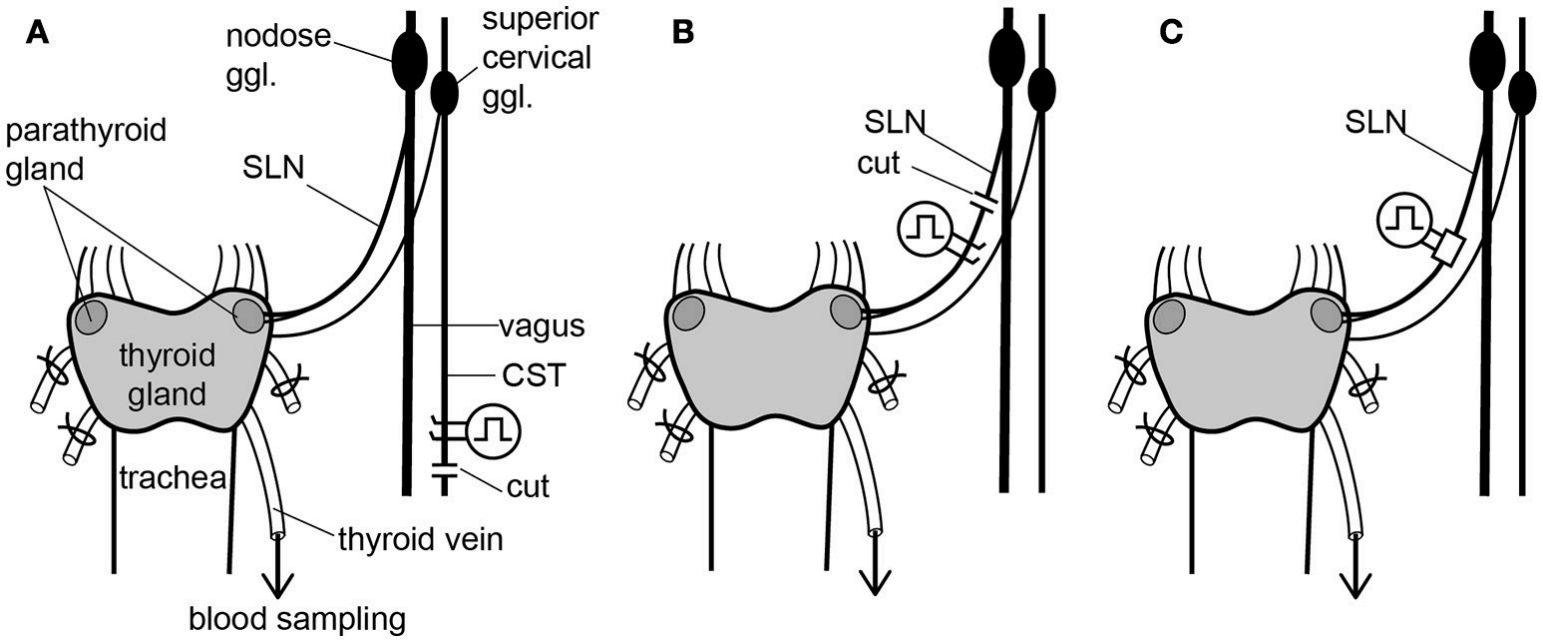
Schematic diagram of the sympathetic (cervical sympathetic trunk: CST) and parasympathetic (superior laryngeal nerve: SLN) innervation of thyroid and parathyroid glands and the arrangements of stimulating electrodes in the three experimental groups. Electrical stimulation was delivered bilaterally to either the CSTs or the SLNs. Only the left CST and SLN are drawn for clarity, but experimental preparations were stimulated bilaterally. **(A)** Stimulation of cut peripheral CST ends by hook electrodes. **(B)** Stimulation of cut peripheral SLN ends by hook electrodes. **(C)** Stimulation of intact SLNs by cuff electrodes.

To stimulate sympathetic efferent nerve fibers innervating the thyroid and parathyroid glands, the CSTs in the neck were cut bilaterally as caudal as possible. The peripheral (rostral) ends of these nerves were placed on bipolar, platinum-iridium wire electrodes (Figure 1A). To stimulate the parasympathetic efferent nerve fibers innervating thyroid and parathyroid glands, the SLNs were cut bilaterally at a site close to the nodose ganglia. The peripheral ends of these nerves were placed on bipolar, platinum-iridium wire electrodes (Figure 1B). Based on stimulus parameters known to be supramaximal for all nerve fibers, the stimulus intensity and pulse duration were kept constant at 10 V and 0.5 ms, respectively (Ito et al., 1987).

To stimulate intact SLNs, we used cuff electrodes (AirRay research Micro Cuff Tunnel, CorTec Freiburg, Germany, internal diameter 200 μm) (Figure 1C). After placing cuff electrodes ~8 mm central from the thyroid gland, we tested single 0.5-ms pulse stimulations of various current intensities to the right and left SLN to determine the threshold intensity (T) that evoked a visible twitch of the cricothyroid muscle under a microscope. The stimulus intensity and pulse duration were kept constant at twice the threshold (2T) intensity and 0.5 ms, respectively. In preliminary experiments designed to record compound action potentials, we confirmed that the threshold intensity for unmyelinated fibers was >10T, and 10 V (>40T) was supramaximal for activating all unmyelinated fibers in both the CST and SLN. By measuring thyroid gland blood flow using a laser Doppler flowmeter, stimulation of the cut peripheral end of the SLN required stimuli >10T to induce vasodilation, whereas stimulation of the intact SLN with 2T was sufficient to produce nearly maximum vasodilation in the thyroid gland.

Stimulation was applied with rectangular pulses using an electronic stimulator (SEN-7203; Nihon Kohden) and isolators (SS-202J; Nihon Kohden). The nerves were kept in warm liquid paraffin to protect them from drying. Frequencies ranging from 0.5 to 40 Hz were tested in each rat. The order of stimulus frequency was randomized to ensure the effects were not related to bleeding during blood sampling.

### Collecting blood samples

The thyroid gland was exposed from the ventral side. A thin polyethylene catheter (outer diameter of tip: ~0.4 mm) was inserted into one branch of the thyroid veins. All other remaining venous branches at the right and left sides were tied with thin threads, as previously described (Ito et al., 1987; Kurosawa et al., 1988; Hotta et al., 1991). Another catheter (outer diameter: 1 mm) was inserted into a subclavian vein. The animals were heparinized (200 IU/kg, i.v.). The two catheters were connected via a silicone tube until the start of thyroid venous blood sampling. After waiting at least 30 min under resting conditions, a thyroid venous blood sample was collected in an ice-chilled polyethylene tube through the thyroid venous catheter. We collected 13–16 consecutive thyroid venous blood samples (200–250 μl each) during each experiment. In each rat, we applied four to five different stimulations. In each stimulation, three blood samples were taken: before, during, and after stimulation. The dead volume (~30 μl) of the thyroid venous catheter at the onset of each stimulus was collected in a capillary tube to measure the hematocrit. The stimulus was maintained until the collecting volume reached 200–250 μl.

The systolic blood pressure was constantly maintained above 80 mmHg by infusing 4% Ficoll PM70 in heparinized saline solution containing 10 mM bicarbonate through a femoral venous catheter during thyroid venous blood sampling at a speed of 3.0 ml/h (heparin: 200 IU/kg/h). Blood samples were centrifuged within 30 min of collection at 3,000 rpm for 15 min at 4°C. Ethylene diamine tetraacetate disodium was added to the collected plasma samples (2–3 mg/ml plasma). The samples were frozen and stored at −20°C until use. The thyroid venous plasma flow rate was calculated from the plasma volume of the thyroid venous blood sample and the sampling time.

### Measurement of CT, PTH, T3, and T4

The concentrations of immunoreactive CT (iCT), PTH (iPTH), T3 (iT3), and T4 (iT4) in thyroid venous plasma were measured by ELISA. We used kits for iCT (rat CT ELISA kit, MBS703165, MyBioSource, San Diego, USA), iPTH (rat intact PTH ELISA kit, 60–2500, Immutopics, San Clemente, USA), iT3 (rat free T3 ELISA kit, CUSABIO, CSB-E05076r, Baltimore, USA), and iT4 (general free T4 ELISA Kit, Cloud-Clone Corp, CEA185Ge, Katy, TX, USA). Thawed plasma samples were centrifuged for 2 min at 4,000 rpm at 4°C before use. For each kit, we confirmed generation of a displacement curve by serial dilution of the thyroid venous plasma and determined the dilution rate (3–10 times). The secretion rate of each hormone was calculated from its concentration in the thyroid venous plasma and the flow rate of thyroid venous plasma, as previously described (Ishii et al., 1968).

### Statistical analysis

Values are expressed as the mean ± standard error. Values obtained before and during stimulation at each frequency was compared by a paired-*t* test. A statistical analysis of basal values from three different conditions (CSTs cut, SLNs cut, and nerves intact) was performed using a one-way factorial ANOVA followed by Dunnett's multiple comparison test. The statistical significance was set at 5%.

## Results

The basal secretion rates of iCT, iPTH, iT3, and iT4 without stimulation in three different experimental groups, i.e., CSTs cut, SLNs cut, and nerves intact, are summarized in Table 2. The basal secretion rates of these hormones varied in each experiment but were stable in individual animals for 2–3 h during the experiment. Hematocrit values gradually decreased but were consistently above 40% in all rats. The threshold (1T) was 2–30 μA in all test rats. To collect the appropriate volume for measurement of the four different hormones, the duration of stimulation varied between 4 and 11 min, depending on the blood flow (15–70 μl/min).

**Table 2:** Summary of basal hormonal secretion rates in the three experimental groups.

**Hormones**	**CSTs cut**	**SLNs cut**	**Nerves intact**
iCT (pg/min)	0.98 ± 0.18^**^	0.50 ± 0.11	0.37 ± 0.07
iPTH (pg/min)	105 ± 49	55 ± 31	28 ± 19
iT3 (fmol/min)	0.109 ± 0.020	0.060 ± 0.008	0.066 ± 0.009
iT4 (pg/min)	0.188 ± 0.072	0.116 ± 0.018	0.130 ± 0.035
Thyroid venous plasma flow (μl/min)	24 ± 5^*^	13 ± 2	13 ± 1

*Each value indicates mean ± standard error*.

* *p < 0.05*,

** *p < 0.01, vs. corresponding value in intact nerves group*.

*iCT, immunoreactive calcitonin; iPTH, immunoreactive parathyroid hormone; iT3, immunoreactive 3,3′,5-triiodothyronine; iT4, immunoreactive thyroxine*.

### iCT secretion

#### Basal levels

Basal secretion rates of iCT under resting conditions without nerve stimulation ranged from 0.1 to 1.5 pg/min [CSTs cut, 0.98 ± 0.18 pg/min (*n* = 6); SLNs cut, 0.50 ± 0.11 pg/min (*n* = 6); and nerves intact, 0.37 ± 0.07 pg/min (*n* = 7)] (Table 2). The iCT basal secretion rate was significantly higher in rats with cut CSTs than in rats with intact nerves (*p* = 0.005). However, iCT secretion did not significantly differ between rats with cut SLNs and rats with intact nerves.

#### Response to nerve stimulation

**Figure 2:**
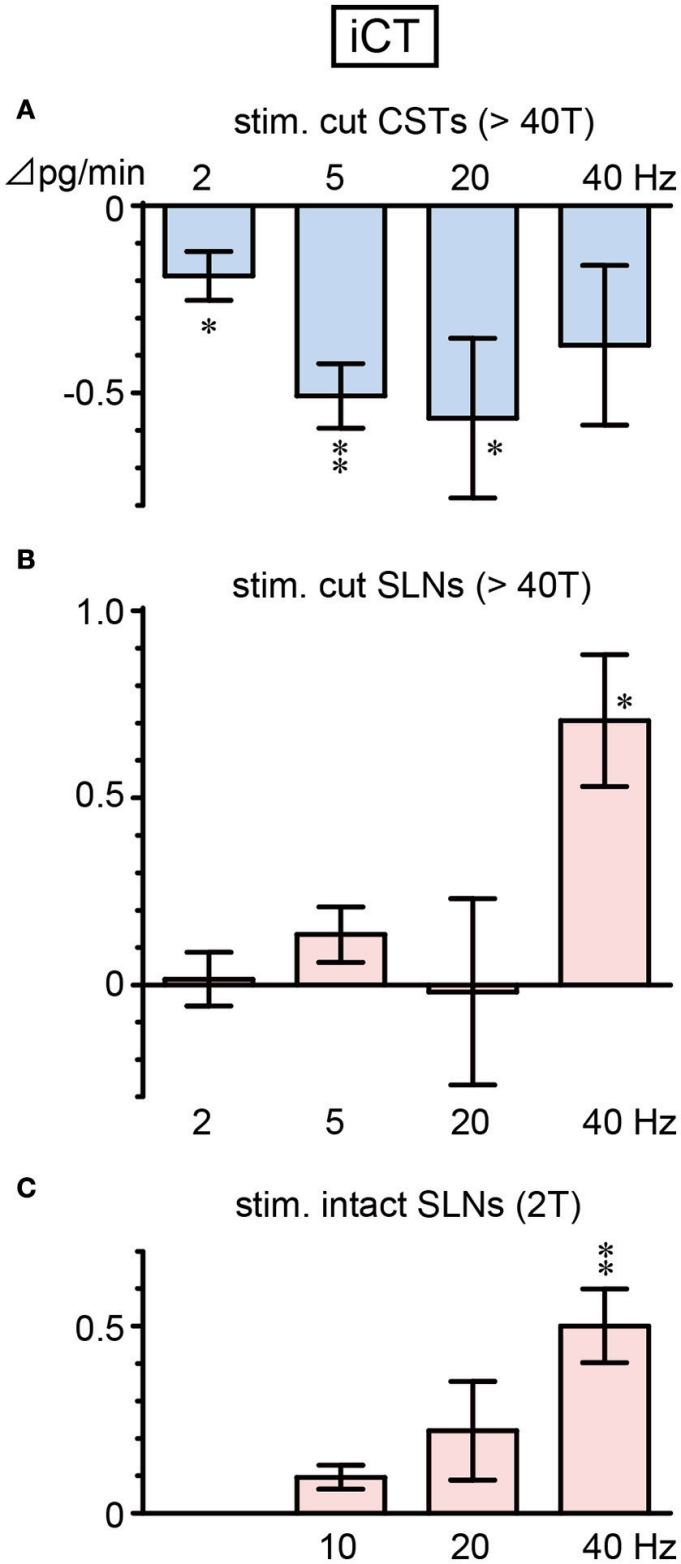
Changes in iCT secretion rate during electrical stimulation of cut CST **(A)**, cut SLN **(B)**, and intact SLN **(C)** at various stimulus frequencies. X-axis: frequency of stimulation. Y-axis: changes in iCT secretion rate from prestimulus control values. Columns and vertical bars show the mean ± standard error. Significant differences were determined by comparison with the prestimulus control values (^*^p < 0.05, ^**^p < 0.01).

iCT secretion decreased during CST stimulation and increased during SLN stimulation, depending on the stimulus frequency (Figure 2). A significant decrease in iCT secretion was observed when CSTs were stimulated at 2, 5, and 20 Hz (*p* = 0.036, 0.002 and 0.045, respectively, Figure 2A). However, this response was attenuated by stimulation at a higher frequency of 40 Hz. In contrast, stimulation of cut SLNs at 40 Hz increased iCT secretion (*p* = 0.010), whereas no significant change was observed at 2–20 Hz (Figure 2B). Similarly, stimulation of intact SLNs also produced the largest increase in iCT secretion at 40 Hz (*p* = 0.004). Stimulation of intact SLNs did not significantly increased iCT secretion at 10 and 20 Hz (Figure 2C).

**Figure 3:**
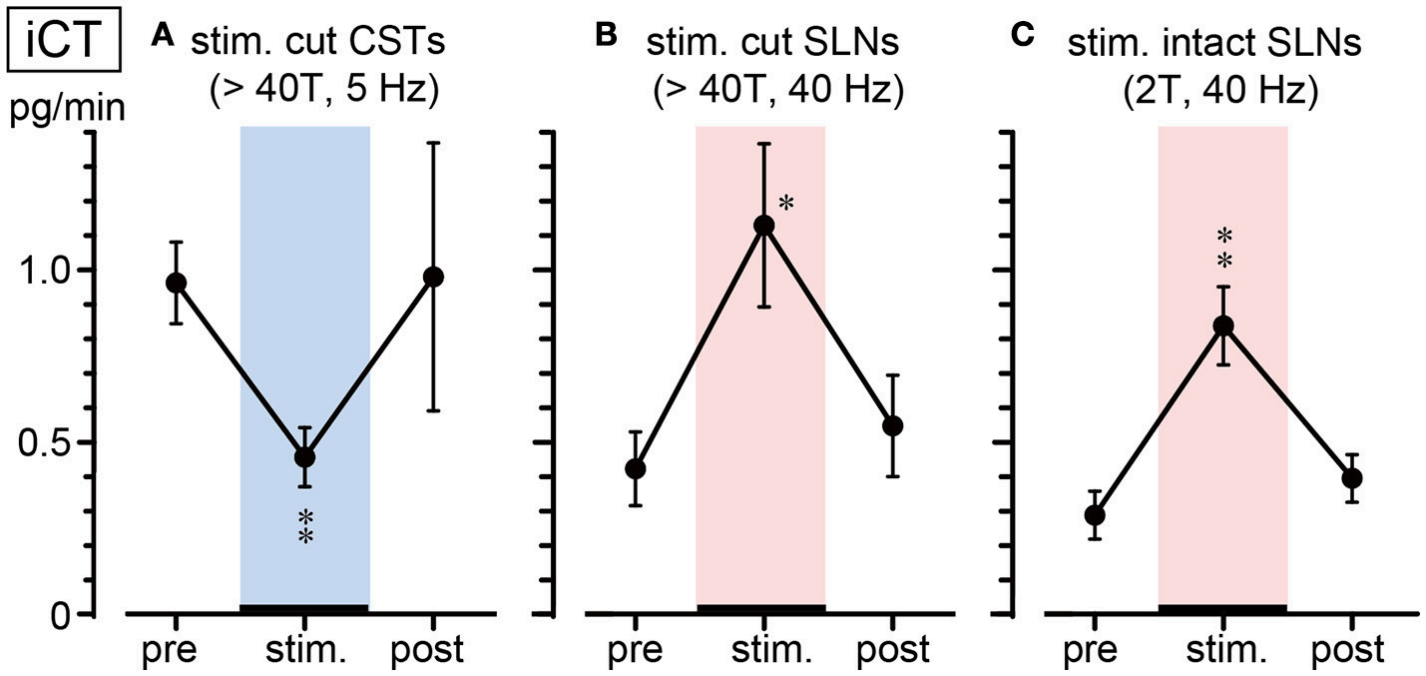
Summary of iCT secretion rate during electrical stimulation of cut CSTs **(A)**, cut SLNs **(B)**, and intact SLNs **(C)**. Circles and vertical bars show the mean ± standard error. Significant differences were determined by comparison with the prestimulus values (^*^p < 0.05, ^**^p < 0.01).

Figure 3 summarizes the changes in iCT secretion before, during, and after stimulation at representative frequencies. Stimulation of cut CSTs at 5 Hz significantly reduced iCT secretion to ~50% of the prestimulus value (0.96 ± 0.12 to 0.46 ± 0.09 pg/min, 6 trials in 6 rats; *p* = 0.002, Figure 3A). However, SLN stimulation at 40 Hz of either the cut peripheral end (0.42 ± 0.11 to 1.13 ± 0.24 pg/min, 6 trials in 6 rats; *p* = 0.010, Figure 3B) or intact nerve (0.32 ± 0.06 to 0.84 ± 0.11 pg/min, 7 trials in 7 rats; *p* = 0.004, Figure 3C) significantly increased iCT secretion to ~260–270% of the prestimulus value. All values returned to the prestimulus level after the cessation of stimulation.

### iPTH secretion

**Figure 4:**
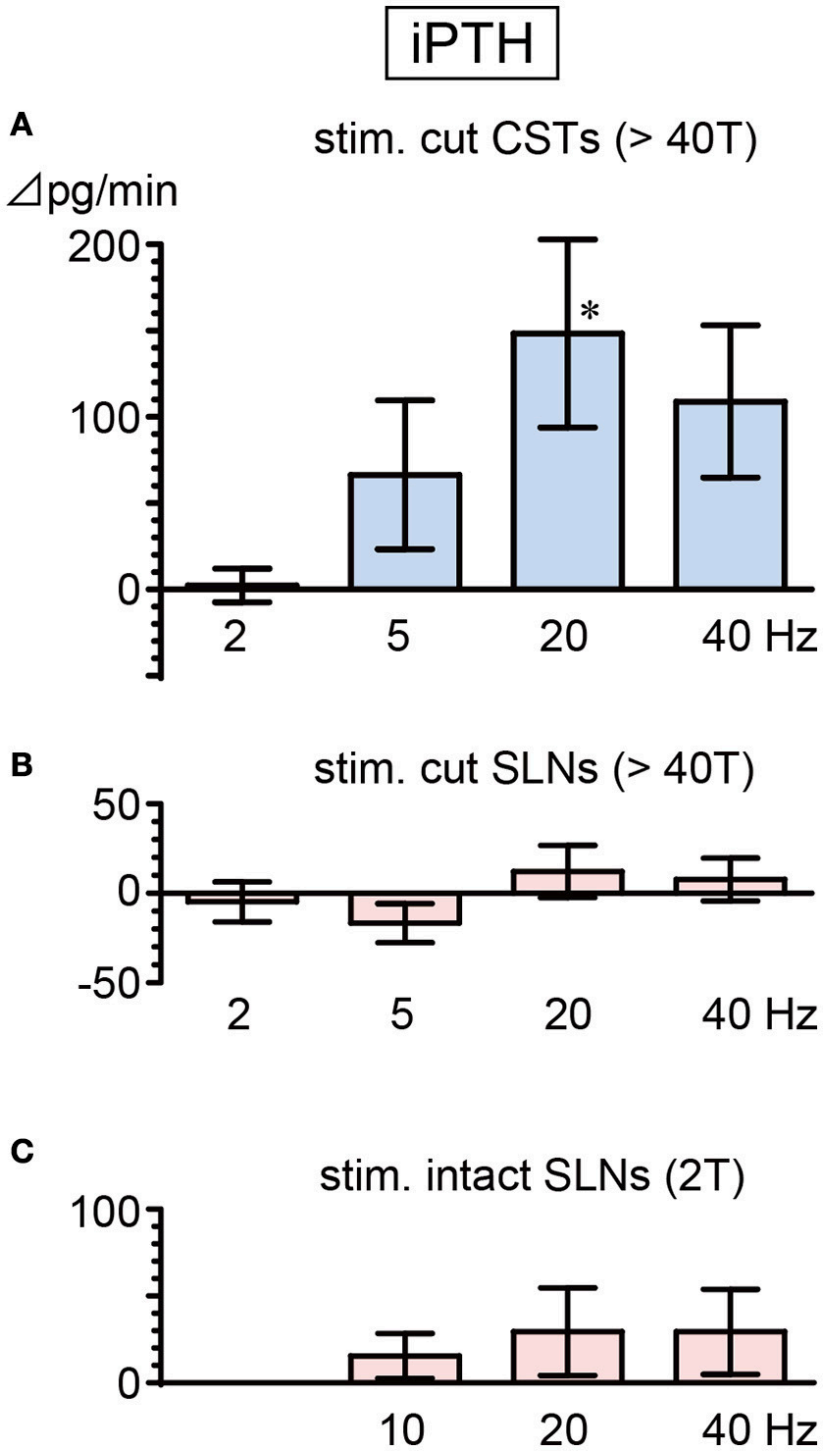
Changes in iPTH secretion rate during electrical stimulation of cut CST **(A)**, cut SLN **(B)**, and intact SLN **(C)** at various stimulus frequencies. X-axis: frequency of stimulation. Y-axis: changes in iPTH secretion rate from prestimulus values. Columns and vertical bars show the mean ± standard error. Significant differences were determined by comparison with the prestimulus control values (^*^p < 0.05).

Basal secretion rates of iPTH were not significantly different between cut CSTs, cut SLNs, and intact nerves (Table 2). In contrast to the observed reduction in iCT secretion, iPTH secretion increased during CST stimulation. iPTH secretion was enhanced by stimulation at higher frequencies but not affected by stimulation at 2 Hz. A significant increase in iPTH secretion, peaking at 240% of the prestimulus control level, was observed at 20 Hz (105 ± 49 to 253 ± 94 pg/min, 6 trials in 6 rats; *p* = 0.042, Figure 4A). The level of iPTH secretion returned to prestimulus levels after the cessation of stimulation. iPTH secretion was not significantly affected by stimulation of cut or intact SLNs at any frequencies (Figures 4B,C).

### IT3 and IT4 secretion

**Figure 5:**
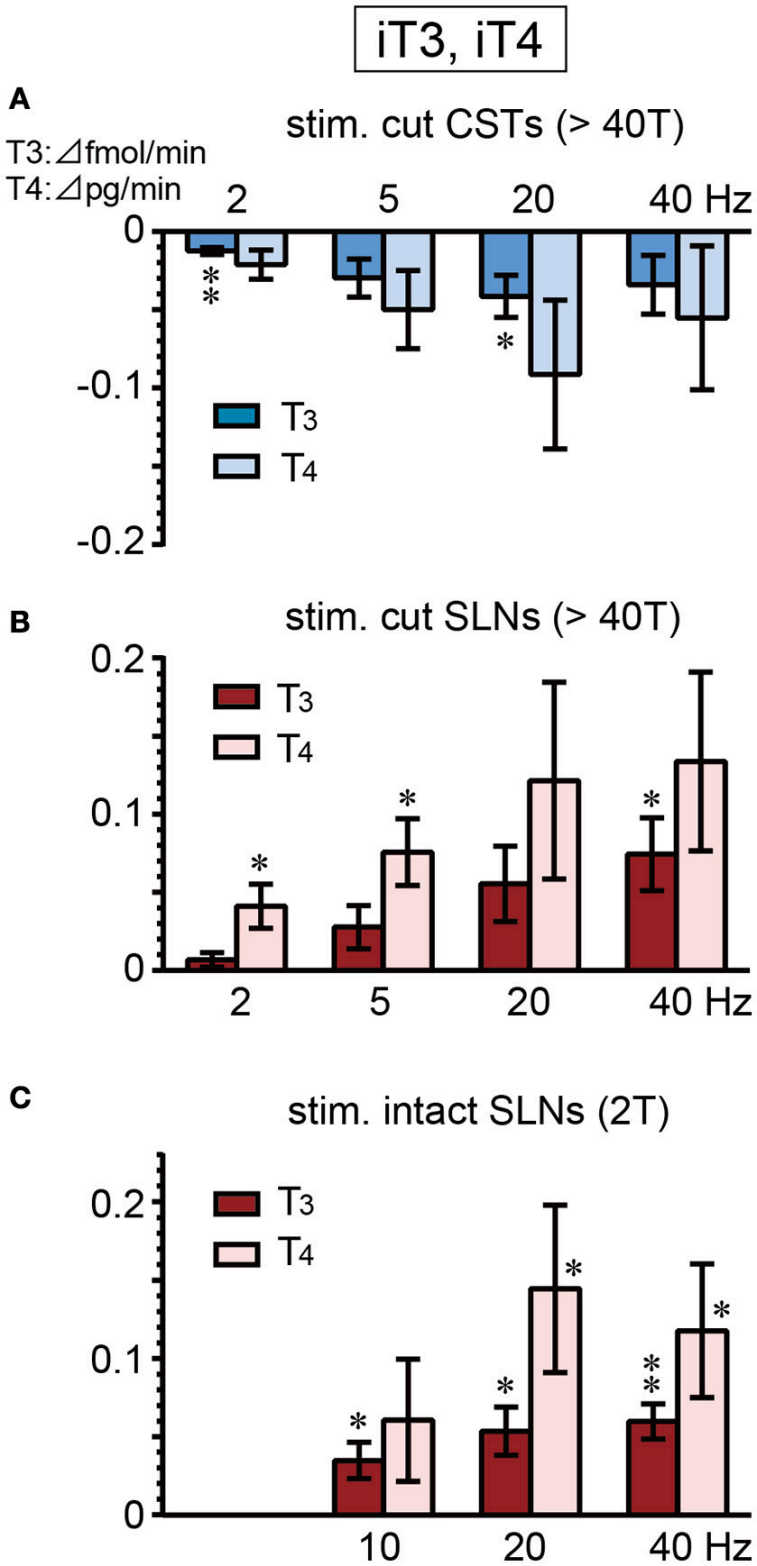
Changes in iT3 and iT4 secretion rate during electrical stimulation of cut CST **(A)**, cut SLN **(B)**, and intact SLN **(C)** at various stimulus frequencies. X-axis: frequency of stimulation. Y-axis: changes in iT3 and iT4 secretion rate from prestimulus control values. Columns and vertical bars show the mean ± standard error. Significant differences were determined by comparison with the prestimulus control values (^*^p < 0.05, ^**^p < 0.01).

There were no significant differences in the basal secretion of iT3 or iT4 between cut CSTs, cut SLNs, and intact nerves (Table 2). CST stimulation at 2–40 Hz decreased iT3 and iT4 secretion (Figure 5A), and significant changes were observed at 2 and 20 Hz for iT3. The decrease in iT3 (0.115 ± 0.015 to 0.073 ± 0.010 fmol/min, 6 trials in 6 rats; *p* = 0.027) at 20 Hz reached 60% of the prestimulus levels. Electrical stimulation of SLNs significantly increased iT3 secretion at 40 Hz for cut SLNs (Figure 5B) and at 10, 20, and 40 Hz for intact SLNs (Figure 5C). The increased levels of iT3 at 40 Hz reached 240% for cut SLNs (0.054 ± 0.011 to 0.128 ± 0.022 fmol/min, 6 trials in 6 rats; *p* = 0.025) and 190% for intact SLNs (0.063 ± 0.010 to 0.122 ± 0.020 fmol /min, 7 trials in 7 rats; *p* < 0.002). Electrical stimulation of SLNs significantly increased iT4 secretion at 2 and 5 Hz for cut SLNs (Figure 5B) and at 20 and 40 Hz for intact SLNs (Figure 5C). The increased levels of iT4 at 40 Hz reached 240% of the prestimulus level for cut SLNs (0.10 ± 0.02 to 0.24 ± 0.05 pg/min, 6 trials in 6 rats; *p* = 0.067) and 180% for intact SLNs (0.14 ± 0.04 to 0.25 ± 0.07 pg/min, 7 trials in 7 rats; *p* = 0.033).

### Systemic blood pressure and thyroid venous plasma flow

Systemic arterial blood pressure increased in response to stimulation of intact SLNs but was not affected by stimulation of cut CSTs or cut SLNs. The significant increase in blood pressure was observed at 20 Hz. The mean increase in blood pressure during stimulation of intact SLNs with a 0.5-ms duration and 2T intensity (4–60 μA) at 20 Hz was 14 ± 4 mmHg (19% of prestimulus levels, *p* < 0.008). At 40 Hz stimulation the blood pressure increased transiently, but the mean changes during stimulation were not significantly different from prestimulus levels.

The basal thyroid venous plasma flow rate under resting conditions was 24 ± 5 μl/min, 13 ± 2 μl/min, and 13 ± 1 μl/min in rats with cut CSTs, cut SLNs, and intact nerves, respectively (Table 2). The flow rate was significantly higher in rats with cut CSTs than rats with intact nerves (*p* = 0.027). We confirmed the previous report (Ito et al., 1987) of a frequency-dependent decrease and increase in thyroid venous blood flow during stimulation of cut CSTs and cut SLNs, respectively. The thyroid venous plasma flow rate significantly decreased by 4 ± 1 μl/min, 7 ± 2 μl/min, 10 ± 4 μl/min, and 8 ± 4 μl/min during stimulation of cut CSTs at 2, 5, 20, and 40 Hz, respectively (*p* < 0.047), significantly increased by 2 ± 1 μl/min, 7 ± 2 μl/min, 12 ± 5 μl/min, and 15 ± 4 μl/min during stimulation of cut SLNs at 2, 5, 20, and 40 Hz, respectively (*p* < 0.028), and significantly increased by 7 ± 2 μl/min, 10 ± 2 μl/min, and 13 ± 2 μl/min during stimulation of intact SLNs at 10, 20, and 40 Hz, respectively (*p* < 0.012).

## Discussion

### Role of sympathetic and parasympathetic efferent activation

Our results demonstrate that the electrical stimulation of parasympathetic SLNs increases iCT secretion from the thyroid gland in rats. Similar effects on iCT secretion were observed in the ultimobranchial body during stimulation (1 ms, 20 V, 10 Hz for 17–43 min) of a cut peripheral portion of the vagus nerve in geese (Care and Bates, 1973). In our study, an increase in iCT secretion was observed only during stimulation of SLNs at 40 Hz, which was the highest frequency tested. This finding suggests that extreme excitation of parasympathetic efferent nerve fibers increases CT secretion. The selective secretion of iCT at 40 Hz contrasted with the significant increase in thyroid venous plasma flow rate following SLN stimulation at 2–40 Hz. Therefore, increased iCT secretion appears to be independent of changes in flow rate and is likely to be controlled by the direct action of transmitters released from parasympathetic nerve terminals on calcitonin secretory cells. A previous study reported that a cholinergic mechanism plays a major role in increasing thyroid venous blood flow during low frequency stimulation of the SLN, whereas a non-cholinergic mechanism may contribute to the increase in thyroid venous blood flow at higher frequencies of stimulation (Ito et al., 1987). In accordance with this finding, secretion of the parasympathetic co-transmitter vasoactive intestinal polypeptide (VIP) was markedly increased during SLN stimulation at 40 Hz (Ito et al., 1987). *In vitro* studies showed that VIP stimulated iCT secretion in rats (Ahrén et al., 1983), whereas cholinergic stimulation had no consistent effect (Sorenson et al., 1983; Gilgenkrantz et al., 1991). Taken together, these findings suggest that stimulation of parasympathetic SLNs at 40 Hz may stimulate CT secretion via VIPergic mechanisms. This hypothesis can be tested in future studies using cholinergic and VIPergic antagonists. Maximal vagus efferent nerve discharge can be reached at 40–50 Hz (Jewett, 1964; McAllen and Spyer, 1978). However, physiological activation typically consists of short bursts at a higher frequency. Therefore, it may be of value to test the effect of burst stimulation of the SLN.

**Figure 6:**
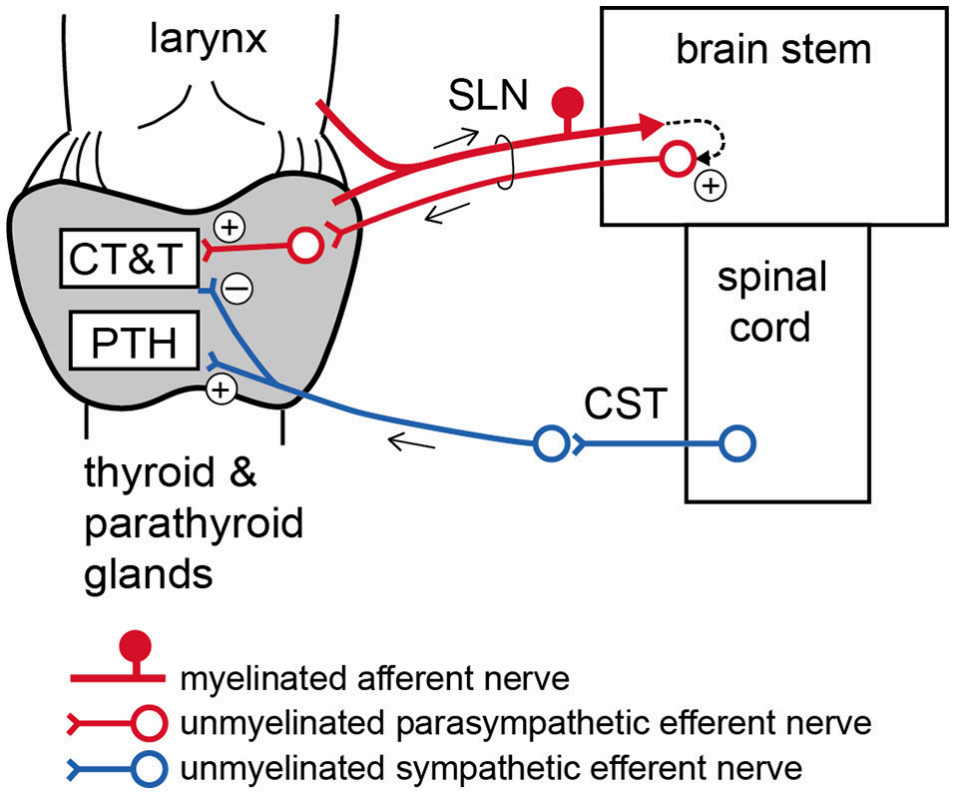
Schematic diagram of proposed autonomic nervous regulation of the thyroid and parathyroid glands. CT, calcitonin; T, thyroid hormone; PTH, parathyroid hormone.

Contrary to the increase in iCT during SLN stimulation, iCT secretion was reduced following sympathetic CST stimulation. This demonstrates an antagonistic action of parasympathetic and sympathetic nerves on CT secretion (Figure 6). Sympathetic-mediated inhibition appears to be tonically active because basal iCT secretion was significantly higher in cut CSTs than intact nerves (Table 2). This finding is in accordance with a previous report suggesting that spontaneous activity in preganglionic single fibers projecting to the superior cervical ganglion in the rat ranged from 0.2 to 3.5 imp/s (Bartsch et al., 2000).

Unlike the decrease in iCT secretion, CST stimulation at 20 Hz increased iPTH secretion. Therefore, the excitation of sympathetic efferent nerve fibers innervating the thyroid and parathyroid glands appears to have opposite effects on the secretion of these two calcium-regulating hormones. PTH and CT play important roles in calcium homeostasis through their actions on osteoblasts (bone forming cells) and osteoclasts (bone resorbing cells), respectively (Carter and Schipani, 2006). Thus, these sympathetic-mediated changes in CT and PTH secretion may cause hypercalcemia and bone loss.

In contrast to the significant increase in iPTH secretion during sympathetic CST stimulation in the present study, electrical stimulation (1 ms, 50 V, 20 Hz, for 10 min) of the right cervical vagosympathetic trunk had no significant effect on the iPTH concentration measured in the systemic blood of dogs (Heath et al., 1985). This discrepancy could be explained by differences in the species or the method of hormonal secretion measurement. Changes in hormonal secretion detected from the vein draining endocrine glands are not necessarily reflected in the systemic blood concentration [e.g., iodide output from a dog's thyroid gland (Ishii, 1960) or estradiol secretion from a rat's ovary (Uchida et al., 2012) after autonomic nerve stimulation]. In the present study, we directly measured hormonal secretion into the thyroid venous blood, thereby providing the first evidence of the neural regulation of calcium regulatory hormone secretion from the thyroid and parathyroid glands.

### Reflex changes in hormonal secretion by activation of parasympathetic myelinated afferents

The present results demonstrate that low intensity electrical stimulation of intact SLNs produces similar changes in iCT, iT3, and iT4 secretion as the supramaximal intensity stimulation of SLN efferents. This finding suggests that excitation of myelinated afferent nerve fibers in the SLN may polysynaptically connect to unmyelinated parasympathetic efferents in SLNs via the central nervous system (Figure 6). The stimulation of intact SLNs increased systemic blood pressure, similar to stimulation of the cut central end of myelinated SLN fibers in urethane-anesthetized rats (Hanamori et al., 1996). Therefore, excitation of afferent SLNs produces reflex excitation of sympathetic efferent vasoconstrictor fibers regulating systemic blood vessels. However, the response of the thyroid and parathyroid glands produced by stimulation of intact SLNs, including secretion of iCT, iPTH, iT3, and iT4 and the thyroid venous plasma flow rate, was similar to parasympathetic efferent stimulation but not sympathetic efferent stimulation. The reflex activation of thyroid-innervating autonomic efferents by stimulation of SLN afferents appears to predominantly occur through parasympathetic rather than sympathetic nerves.

The SLN contains various afferent fibers, such as mechanoreceptors and chemoreceptors, from the laryngeal mucosa (Coleridge and Coleridge, 1997). Sensory innervation of the thyroid itself has been shown in histological studies, although the functional role of this innervation has not been determined (Grunditz et al., 1996). Afferent axons in the SLN reach the nucleus of the solitary tract, while parasympathetic efferents in the SLN originate from the dorsal motor nucleus of the vagus and the nucleus ambiguous. These central connections are exclusively ipsilateral (Pascual-Font et al., 2011). Further investigation is necessary to determine what kind of natural stimulation and which modality of myelinated afferent fibers in the SLN trigger the reflex response of hormonal secretion from the thyroid gland and which brain areas increase SLN efferent nerve activity.

The response induced by low intensity stimulation of intact SLNs has clinical neuromodulation potential. The 2T intensity (4–60 μA) used to stimulate intact SLNs in the present study was much lower than the intensity used clinically for vagal neuromodulation (Handforth et al., 1998; DeGiorgio et al., 2005). In rats, low intensity stimulation of the intact SLN mediates activation of parasympathetic efferents via a reflex, whereas a medical application of this low intensity stimulation may directly activate myelinated preganglionic parasympathetic fibers in humans. Regardless of whether activating afferents or efferents, our results suggest that low intensity (2T, 0.5 ms) stimulation of intact SLNs increase the secretion of CT, T3, and T4, but not PTH (at 40 Hz). Therefore, stimulation of SLNs at 40 Hz may help to prevent bone loss in osteoporosis by increasing CT secretion.

The traditional pharmacological treatments for osteoporosis have various problems, such as poor pharmacokinetic properties (e.g., short half-life of salmon or eel calcitonin), frequent occurrence of adverse effects (e.g., bisphosphonates), and variability in patient compliance (Yamauchi et al., 2003; Asafo-Adjei et al., 2016; Tabatabaei-Malazy et al., 2017). Neuromodulation therapies have been used for decades, including pacemakers, defibrillators, and deep-brain stimulation, and vagus nerve stimulation has been used to treat conditions such as epilepsy and rheumatoid arthritis; however, many of these approaches do not target specific cells/circuits (Famm et al., 2013). Implantable devices, which can be attached to peripheral nerves anywhere in the viscera, are used to treat hypertension and sleep apnea. With anatomical and technological developments in many areas, such as neural interfacing technology, there is now greater potential for high precision approaches to treat patients through specific neuromodulation (Birmingham et al., 2014). The data generated in this study demonstrates that specific intervention and stimulation of the SLN can selectively modify hormonal release from the parathyroid/thyroid glands, which has a potential application in patients, e.g., increasing endogenous calcitonin in cases of osteoporosis.

## Conclusion

In conclusion, our results demonstrate that sympathetic and parasympathetic efferent fibers modulate the secretion of calcitonin and thyroid hormone antagonistically from the thyroid gland, while the sympathetic nerve promotes PTH secretion from the parathyroid gland. Furthermore, we showed that stimulation of SLN myelinated afferent fibers promotes calcitonin and thyroid hormone secretion from the thyroid glands, potentially by reflex excitation of the parasympathetic efferent fibers. These findings suggest a possible novel bioelectronic medicine application of neuromodulation therapy for altering calcitonin levels, increasing bone density, decreasing bone resorption, and/or increasing bone formation.

## Author contributions

HH and HS contributed to study design, data acquisition, data analysis, data interpretation, and manuscript writing. AO contributed to data acquisition, data analysis, and manuscript revising. PM and AS contributed to study design, data interpretation, and manuscript writing. All authors approved the final version of the manuscript and agreed to be accountable for all aspects of the work in ensuring that questions related to the accuracy or integrity of any part of the work are appropriately investigated and resolved.

### Conflict of interest statement

The authors of this manuscript have a patent application on a Neuromodulation device.
